# Supplementary material for: Perioperative pupil size in low-energy femtosecond laser-assisted cataract surgery
Source: PLoS One. 2021 May 17;16(5):e0251549. doi: 10.1371/journal.pone.0251549 (PMC8128224; doi:10.1371/journal.pone.0251549)
Supplement: Attachment — Submitted filename: R2_Response to Reviewers.docx [file pone.0251549.s002.docx]

**Response to Reviewers**

Reviewer #1: Additional minor recommendations and comments:

Line 253: “Although all three laser platforms […]” can be the start of

a new paragraph.

- A new paragraph was included as recommended.

Line 293: If subjects were given a dose of NSAID before surgery, I’m

not sure how it can be concluded that the decrease in inflammatory

reaction results from the use of lower pulse energy FSL and not from

the use of pre-operative NSAIDs. Authors should confirm whether this

was in fact a conclusion of the cited study and/or clarify this

statement.

- As stated in the section “surgical technique” no NSAID was given to any patient before surgery in our study.
- With regards to Schwarzenbacher’s paper we acknowledge the anti-inflammatory effect of preoperative NSAID; We believe the authors’ conclusion is based on the fact that the measured inflammatory cytokine levels were lower than reported in other studies using other laser platforms. Our wording is exactly the conclusion of the cited study. Nevertheless, we added this issue to the discussion (Line 296-297)

Line 323-324: This is still an orphaned sentence and does not appear

to have been modified from the original. I’m uncertain as to its

relevance, but it can probably be incorporated into another paragraph

earlier in the manuscript if you choose to keep it.

- The sentence was deleted.

Line 342: There is no reason to abbreviate “including”. The semicolon

should be a period.

- The changes were made as recommended.

Lines 339-343: I would advise caution with this broad assumption as it

seems to directly contradict your statement on the pupillary effects

of peribulbar anesthesia in Lines 306-308.

- We revised the wording and hope it is more suitable now. Lines 342-346

Reviewer #2:

It is not accurate to say peribulbar anesthesia does not

affect pupil size. This is a fatal flaw of the manuscript. If 46% of

cases in Germany done under peribulbar, then you should review the

results of topical as well. Not sure why those with topical anesthesia

were excluded from the study.

- The authors do not claim peribulbar anesthesia does not affect the pupil size. We just believe the results of our study would have been similar under other anesthetic methods. Nevertheless, we revised the wording and hope it is more suitable (Lines 342-346). The authors perform almost all cataract surgeries under peribulbar anesthesia, thus the paper is solely representing those cases.
